# CBCT evaluation of healing after TMJ ankylosis surgery: A prospective analysis

**DOI:** 10.6026/973206300214873

**Published:** 2025-12-15

**Authors:** Kavita Sharma, N. Sriram Choudary, Aayush Malhotra, Gokul Venkateshwar, Nitin Pratap Shishodia, Haripriya Yarlagadda, Rahul Tiwari

**Affiliations:** 1 Department of Dentistry, Government Medical College Sheopur, Madhya Pradesh, India; 2 Department of Oral & Maxillofacial Surgery, Dhanalakshmi Srinivasan Dental College & Hospital, Siruvachur, Perambalur, Tamil Nadu India; 3 Department of Oral and Maxillofacial Surgery, M. M. College of Dental Sciences and Research, Maharishi Markandeshwar (Deemed-to-be University), Mullana, Ambala, Haryana, India; 4 Department of Oral and Maxillofacial Surgery, D. Y. Patil University School of Dentistry, Nerul, Navi Mumbai, Maharashtra, India; 5 Department of Oral Medicine & Radiology, Seema Dental College & Hospital, Rishikesh, Uttarakhand, India; 6 Department of Oral and Maxillofacial Surgery, Care Dental College and Hospital, Guntur, Andhra Pradesh, India; 7 Department of Oral and Maxillofacial Surgery, RKDF Dental College and Research Centre, Sarvepalli Radhakrishnan University, Bhopal, Madhya Pradesh, India

**Keywords:** Temporomandibular joint ankylosis, cone-beam computed tomography, arthroplasty, postoperative healing, prospective studies

## Abstract

Temporomandibular joint (TMJ) ankylosis is a debilitating disorder marked by fibrous or bony fusion of the joint, resulting in restricted mandibular mobility and impaired oral function. Therefore, it is of interest to assesse healing outcomes following temporomandibular joint (TMJ) ankylosis surgery using cone beam computed tomography (CBCT). Postoperative bone remodeling, joint space and functional recovery were evaluated. Data shows significant improvement in joint morphology and mandibular function, confirming CBCT as a reliable modality for assessing surgical outcomes.

## Background:

Temporomandibular joint (TMJ) ankylosis is a debilitating condition characterized by fibrous or bony fusion of the joint, leading to restricted mandibular movements and impaired function [1]. It may result from trauma, infection, or systemic conditions and often causes facial asymmetry, growth disturbances in children and compromised quality of life [2]. The surgical management of TMJ ankylosis remains challenging, with gap arthroplasty, interpositional arthroplasty and joint reconstruction being the most common techniques [3]. Despite advancements, recurrence and inadequate functional recovery remain key concerns [4]. Cone beam computed tomography (CBCT) has emerged as a valuable imaging modality due to its high-resolution visualization of osseous structures with relatively low radiation exposure [5]. It allows detailed evaluation of postoperative bone remodeling, joint space creation and anatomical changes [6]. By integrating objective imaging with clinical assessment, the research provides evidence-based insights into the effectiveness of surgical interventions [7]. Therefore, it is of interest to report the postoperative healing outcomes of TMJ ankylosis surgery using CBCT and correlate radiological changes with functional recovery.

## Materials and Methods:

A prospective clinical study was conducted on patients diagnosed with TMJ ankylosis and scheduled for surgical intervention. Ethical clearance was obtained and informed consent was taken from all participants. For the selection criteria the included subjects were patients aged 10-40 years; with unilateral or bilateral TMJ ankylosis confirmed clinically and radiographically and had no prior surgical intervention for TMJ ankylosis. The excluded patients were those with systemic bone disorders; those with contraindications to surgery or imaging and those Non-consenting individuals. A total of 30 patients were enrolled, comprising 18 males and 12 females. Patients underwent either gap arthroplasty or interpositional arthroplasty with temporalis muscle/fascia grafts, as per clinical indication. The aim was to restore joint space and mandibular movements. CBCT scans were obtained preoperatively and at 3 months and 6 months postoperatively. Parameters assessed included:Joint space measurement (mm);Condylar morphology and remodeling;Presence of heterotopic bone formation and Symmetry with contralateral joint. Mouth opening (interincisal distance), deviation on opening and pain (VAS score) were recorded at baseline, 3 months and 6 months. Data were analyzed using paired t-tests and ANOVA. A p-value <0.05 was considered statistically significant.

## Results:

Mean age: 22.4 ± 6.3 years. Male: Female ratio = 1.5:1. Etiology: Trauma (70%), infection (20%), idiopathic (10%) The postoperative results demonstrated significant functional recovery in patients undergoing TMJ ankylosis surgery. Interincisal distance improved dramatically from a severely restricted mean of 7.8 mm preoperatively to 28.4 mm at 3 months and further to 32.1 mm at 6 months. Simultaneously, pain scores decreased substantially, from a preoperative mean of 6.8 to 2.1 at 3 months and 1.5 at 6 months. These improvements, both statistically significant, highlight the effectiveness of surgical intervention and postoperative physiotherapy (Table 1 - see PDF). CBCT analysis revealed substantial radiological improvements after surgery. The joint space increased from a negligible 0.9 mm preoperatively to 4.2 mm at 3 months and stabilized at 4.6 mm at 6 months, indicating durable space maintenance. Remodeling patterns showed a shift toward smoother condylar surfaces, increasing from 14 to 22 cases, while irregular surfaces reduced from 10 to 5. Early bone fusion decreased from 6 to only 3 cases. These findings demonstrate progressive structural healing and reduced recurrence risk (Table 2 - see PDF).

## Discussion:

This prospective analysis highlights the effectiveness of TMJ ankylosis surgery in restoring function and morphology, with CBCT playing a pivotal role in postoperative assessment. The marked improvement in mouth opening corroborates earlier studies emphasizing surgical release as the cornerstone of management [8]. Maintenance of interincisal distance up to 6 months reflects the efficacy of physiotherapy compliance and surgical technique [9]. CBCT analysis demonstrated successful creation of joint space, with stability observed during follow-up. Similar outcomes were reported in studies where CBCT was used for evaluating postoperative changes in TMJ patients [10]. The gradual remodeling of condylar surfaces underscores bone adaptation and healing, consistent with biological principles of joint recovery [11]. The reduction in irregular bone formation and early fusion rates highlights the effectiveness of interpositional grafts in preventing re-ankylosis [12]. However, recurrence remains a possibility, particularly in pediatric patients due to growth-related remodeling [13]. Pain reduction and improved function align with previous reports of enhanced quality of life following surgical correction [14]. Importantly, CBCT provided superior visualization compared to conventional radiography, offering three-dimensional assessment critical for detecting subtle changes [15].

## Conclusion:

TMJ ankylosis surgery, when combined with postoperative physiotherapy, significantly improves functional outcomes. CBCT serves as a reliable, objective imaging modality for evaluating healing, joint space and bone remodeling. This study reaffirms the role of CBCT in evidence-based postoperative monitoring, aiding in early detection of recurrence and optimizing patient care.
